# Update on shedding and transmission routes of porcine haemotrophic mycoplasmas in naturally and experimentally infected pigs

**DOI:** 10.1186/s40813-021-00229-8

**Published:** 2021-08-26

**Authors:** Julia Ade, Mathias Ritzmann, Christopher Wöstmann, Matthias Eddicks, Sven Reese, Katharina Hoelzle, Ludwig E. Hoelzle, Julia Stadler

**Affiliations:** 1 Clinic for Swine, Centre for Clinical Veterinary Medicine, LMU Munich, Sonnenstr. 16, Oberschleissheim, 85764 Munich, Germany; 2 Institute of Animal Science, University of Hohenheim, Stuttgart, Germany; 3 Institute for Anatomy, Histology and Embryology, LMU Munich, Munich, Germany

**Keywords:** Porcine haemotrophic mycoplasmas, *Mycoplasma suis*, ‘*Candidatus* Mycoplasma haemosuis’, Oral inoculation, Shedding patterns

## Abstract

Horizontal transmission of *Mycoplasma suis* via parenteral exposure during standard practices or through bites during fightings have been identified as key epidemiological routes. However, as knowledge gaps on other potential shedding and transmission routes exist, the present study combines both laboratory experiments and field surveys to gain new insights into the epidemiology of porcine haemotrophic mycoplasmas. Splenectomised pigs were orally inoculated with a *M. suis* field strain and investigated for clinical signs related to infectious anaemia of pigs (IAP) and the presence of *M. suis* in blood, urine and saliva samples by qPCR. All blood samples were negative for *M. suis* and animals did not show obvious clinical signs of IAP throughout the entire study period. Additionally, urine, nasal and saliva samples from sows of conventional piglet producing farms and semen samples from a boar stud revealed no detection of *M. suis* and ‘*Candidatus* Mycoplasma haemosuis’ by qPCR. Thus, the results indicate that blood-independent transmission routes might be of minor relevance under field conditions.

## Background

Haemotrophic mycoplasmas (HMs) are uncultivable bacteria found on the surface of red blood cells (RBCs) of numerous domestic and wild mammals [1]. *Mycoplasma* *suis*, the mostly studied porcine HM species, is considered as the causative agent of infectious anaemia in pigs (IAP), causing important economic losses in pig production [2, 3]. The disease can either occur as an acute, haemolytic anaemia attended by high fever and life-threatening conditions or as a chronic or even subclinical form of disease with mild to moderate anaemia and unspecific clinical signs [2, 3]. Recently, a novel HM species currently named as ‘*Candidatus* (*Ca*.) Mycoplasma haemosuis’ was described in subclinical diseases as well as in accordance with IAP-like signs in pigs in China, Korea and Germany [4–6].

Natural routes of porcine HM transmission remain rather unknown [7–9]. Experimental transmission by intravenous, intramuscular, subcutaneous, intraperitoneal and oral inoculation of *M. suis* containing blood has been successfully performed [7, 10]. However, oral infection experiments were conducted prior to the establishment of specific and sensitive PCR assays using microscopic methods [7]. Thus, the first objective of the study was to demonstrate the possibility of oral infection in experimentally infected pigs compared to a subcutaneous infected control group.

Recently, *M.* *suis* shedding was demonstrated in blood-free excretions (i.e. saliva, urine, nasal and vaginal secretion) [11] suggesting transmission of porcine HMs through direct contact via excretions. However, the investigated excretions originated from experimentally infected pigs with high blood loads [11]. Thus, the second aim of the study was to investigate shedding of porcine HMs (*M.* *suis* and ‘*Ca*. M. haemosuis’) via blood-independent excretions (saliva, urine and semen samples) under field conditions.

## Results

### Clinical and pathological observations during experimental *M. suis* infection

**Fig. 1:**
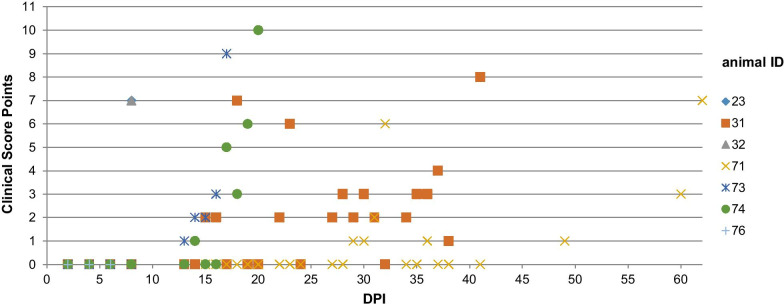
Clinical score points of all seven animals of group B during the course of experimental infection

In the group of orally infected pigs (group A), clinical signs related to IAP were absent in all seven animals until the end of the study on 90 DPI and the clinical score remained at zero points for each animal. In contrast, all subcutaneously infected piglets (group B) showed typical signs of IAP as described by Stadler et al. [12]. Three animals (ID 23, 32, 76) developed fever, apathy, anorexia and skin alterations on 7 and 8 DPI. The determination criteria (i.e. high fever, anorexia, impaired general health) were reached on 8 DPI and the three pigs were humanely euthanized. The four remaining animals showed cyanoses of the ears between13 and 15 DPI. Clinical IAP signs exacerbated in those four remaining animals and euthanasia had to be performed on 17 (ID 73), 20 (ID 74), 41 (ID 31) and 62 (ID 71) DPI, respectively. Clinical score points of group B animals (ID 23, 31, 32, 71, 73, 74, 76) are shown in Fig. 1. During necropsy, none of the seven orally infected pigs (group A) showed macroscopic alterations. Thus, no further microscopic investigation was conducted in those animals. Group B animals (subcutaneously infected) showed various macroscopic (i.e. e. severe icterus of membranes, yellowish discoloration of skin and body fluids, pale musculature) and histopathological lesions (i.e. hyaline thrombi and globules in alveolar vessels, haemosiderin deposits in macrophages, dilatation of lymph vessels, periportal and centrilobular necrosis) as described elsewhere [12].

### Detection of *M. suis* in blood, saliva and urine samples of experimentally infected pigs

*Mycoplasma suis* qPCR remained negative for blood, urine and saliva samples of all group A piglets throughout the entire study period. In group B, positive *M.* *suis* results were obtained from the blood of all animals as described elsewhere [12]. Briefly, on 4 DPI *M.* *suis* was first detected in the blood of three animals (ID 23, 74, 76) and on 6 DPI in all seven animals of this group. Subsequently, *M.* *suis* was permanently present in the blood of group B animals until the individual termination point of the study [12]. *M.* *suis* blood loads varied between 2.2 × 10^3^ and 8.6 × 10^9^ *M.* *suis*/mL blood.

qPCR could also detect *M. suis* in the urine samples of two animals on 8 DPI (ID 73) and on 28 and 48 DPI (ID 71), respectively. *M.* *suis* loads in urine samples varied between 2.40 × 10^4^ and 5.5 × 10^4^ *M.* *suis*/mL urine. In each of the three urine samples, Servotest^®^ 5 + NL stripes showed the presence of RBC residues.

Regarding the saliva samples of group B animals, *M.* *suis* was evident in two animals on 8 DPI (ID 31 + ID 71), and on 15 DPI in one animal (ID 71). Bacterial loads in saliva samples varied between 2.0 × 10^3^ and 5.26 × 10^3^ *M.* *suis*/mL saliva.

*M. suis* loads in blood, saliva and urine samples of group B animals as determined by qPCR are shown in Table 1.

**Table 1:** *Mycoplasma* *suis* quantification of blood, urine and saliva samples determined by qPCR during experimental infection in group B animals (subcutaneously infected animals)

Days post infection	*M. suis*/mL	Animal ID (group B animal)						
		ID 23	ID 31	ID 32	ID 71	ID 73	ID 74	ID 76
2	Blood	Negative	Negative	Negative	Negative	Negative	Negative	Negative
	Urine	Negative	Negative	Negative	Negative	Negative	Negative	Negative
	Saliva	Negative	Negative	Negative	Negative	Negative	Negative	Negative
4	Blood	**2.2** × **10**^**3**^	Negative	Negative	Negative	Negative	**2.0** × **10**^**5**^	**6.0** × **10**^**4**^
	Urine	Negative	Negative	Negative	Negative	Negative	negative	Negative
	Saliva	Negative	Negative	Negative	Negative	Negative	Negative	Negative
6	Blood	**4.6** × **10**^**8**^	**1.1** × **10**^**4**^	**4.2** × **10**^**7**^	**3.6** × **10**^**5**^	**1.9** × **10**^**4**^	**6.6** × **10**^**4**^	**1.1** × **10**^**9**^
	Urine	Negative	Negative	Negative	Negative	Negative	Negative	Negative
	Saliva	Negative	Negative	Negative	negative	Negative	Negative	Negative
8	Blood	**3.6** × **10**^**9**^**†**	**9.2** × **10**^**6**^	**9.6** × **10**^**9**^**†**	**3.0** × **10**^**9**^	**3.0** × **10**^**7**^	**1.8** × **10**^**7**^	**8.6** × **10**^**9**^**†**
	Urine	Negative	Negative	Negative	Negative	**5.5** × **10**^**4**^	Negative	Negative
	Saliva	Negative	**2.0** × **10**^**3**^	Negative	**2.84** × **10**^**3**^	Negative	Negative	Negative
14	Blood		**1.4** × **10**^**8**^		**1.9** × **10**^**7**^	**3.6** × **10**^**8**^	**2.2** × **10**^**7**^	
	Urine		Negative		Negative	Negative	Negative	
	Saliva		Negative		**5.26** × **10**^**3**^	Negative	Negative	
17*	Blood		n.d		n.d	**2.4** × **10**^**9**^**†**	n.d	
	Urine		n.d		n.d	Negative	n.d	
	Saliva		n.d		n.d	Negative	n.d	
20*	Blood		n.d		n.d		**1.3** × **10**^**7**^**†**	
	Urine		n.d		n.d		Negative	
	Saliva		n.d		n.d		Negative	
21	Blood		**1.7** × **10**^**9**^		**2.0** × **10**^**8**^			
	Urine		Negative		Negative			
	Saliva		Negative		Negative			
28	Blood		**5.4** × **10**^**7**^		**4.7** × **10**^**6**^			
	Urine		Negative		**3.5** × **10**^**4**^			
	Saliva		Negative		Negative			
30*	Blood		**7.5** × **10**^**8**^		n.d			
	Urine		Negative		n.d			
	Saliva		Negative		n.d			
35	blood		**4.4** × **10**^**5**^		**7.2** × **10**^**7**^			
	Urine		Negative		Negative			
	Saliva		Negative		Negative			
41*	Blood		**9.2** × **10**^**7**^**†**		n.d			
	Urine		Negative		n.d			
	Saliva		Negative		n.d			
42	Blood				**1.8** × **10**^**7**^			
	Urine				Negative			
	Saliva				Negative			
48	Blood				**1.4** × **10**^**9**^			
	Urine				**2.5** × **10**^**4**^			
	Saliva				Negative			
49*	Blood				**1.9** × **10**^**8**^			
	Urine				Negative			
	Saliva				Negative			
56	Blood				**1.0** × **10**^**4**^			
	Urine				Negative			
	Saliva				Negative			
60*	Blood				**2.9** × **10**^**9**^			
	Urine				Negative			
	Saliva				Negative			
62	Blood				**4.9** × **10**^**7**^**†**			
	Urine				Negative			
	Saliva				Negative			

Bold lines represent a positive detection of *M. suis*

*n.d.* not determined

^*^Additional sampling date due to exacerbation of clinical signs, only clinically affected animals were sampled at this study date

^†^Euthanisation

### Detection of HMs in blood, urine, saliva and semen in field samples

*Mycoplasma suis* was detected in the blood from 61 out of 150 sampled sows while ‘*Ca.* Mycoplasma haemosuis’ was detected in 13 out of the 150 samples. Each of the 13 ‘Ca. M. haemosuis’ positive sows were co-infected with *M. suis*. Blood loads varied from 2.04 to 3.74 × 10^7^ *M.* *suis* per mL blood and from 7.16 × 10^3^ to 4.88 × 10^4^ ‘*Ca. M* *haemosuis’* per mL blood, respectively. All 148 corresponding saliva samples (59 samples of HM blood positive sows) revealed negative qPCR results for both HM species (all samples p < 0.001, power 100%; samples of bacteremic sows p = 0.004, power 100%).

Similarly, all 47 urine samples (16 samples of HM blood positive sows) revealed negative qPCR results for both HM species (all samples p = 0.014, power 99.8%; samples of blood positive sows p = 0.371, power < 0.1%). RBC residues were not detected in any of the urine samples.

No evidence of *M.* *suis and ‘Ca. M. haemosuis’* infections was detected in any of the 183 tested boars from the boar stud as determined by qPCRs.

## Discussion

Up-to-date, transmission of porcine HMs is thought to mainly occur horizontally during zootechnical procedures and ranking fights. Additionally, results from Heinritzi [7] suggested transmission through oral intake of *M.* *suis* containing blood. However, oral infection with *M.* *suis* was not proven in our experimental study due to the absence of clinical signs and *M. suis* in blood samples determined by qPCR throughout the entire study period. Possible explanations for the deviating results might be the different inoculation strains or the higher inoculation dose used by Heinritzi [7] (10 mL containing 5.8 × 10^9^–4.17 × 10^10^ *M.* *suis*/mL) compared to the present experiment (1.5 mL; 2.0 × 10^7^ *M. suis*/mL). However, the inoculation dose chosen by Heinritzi [7] does not represent a realistic scenario for field infections as the mean bacterial blood loads found in piglets and sows in previous qPCR field studies were much lower [13, 14]. Thus, an inoculation dose in accordance with recent studies was chosen in our experiment. Additionally, in contrast to microscopic examination lacking specificity and sensitivity, our results resemble the first investigation of oral transmission routes using up-to-date real-time PCR assays. Similar results were found for the feline HM species ‘*Ca.* M. turicensis’ [15] as oral inoculation with ‘*Ca.* M. turicensis’ containing blood was not successful in cats.

The detection of *M.* *suis* in different secretes and excretes of experimentally infected pigs has raised issues on blood independent HM transmission routes [11]. In accordance with this previous study, *M.* *suis* could also be detected in urine and saliva samples of group B animals (subcutaneously infected) after experimental infection. Despite comparable *M. suis* loads in both studies Dietz et al. [11] found a higher number of animals and samples positive for *M. suis* in urine and saliva. Interestingly, in the previous study of Dietz et al. [11] *M.* *suis* was also present in urine without RBC residues whereas all *M. suis* positive urine samples in our study contained RBC residues. Consequently, no RBC-free secretion in urine was observed in the present study. This might be attributable to the variation of the *M.* *suis* strain in both studies [11], e.g., the inoculation strain used by Dietz et al. [11] displayed an additional cell tropism for endothelial cells [16].

Regarding samples of naturally infected sows, neither *M.* *suis* nor ‘*Ca*. M. haemosuis’ could be identified in urine and saliva samples under field conditions despite the presence of HMs in the corresponding blood samples. This might also be explained by variation of HM strains, higher *M. suis* blood loads in experimental studies [10–12] compared to naturally infected pigs [13, 14], or a higher susceptibility for HM infections due to the splenectomised pig model used for experimental infections [17]. Furthermore, it still has to be scrutinized under experimental conditions if the PCR positive secretes and excretes of the aforementioned experimentally infected animals actually contain infectious organisms.

Despite the very successful application of oral fluid-based testing facilitates for monitoring, surveillance and detection of several pathogens relevant for the swine production [18–24] oral fluids seem not to resemble a suitable diagnostic specimen for the detection of HMs. However, limitations of our study arise from the number of investigated animals and the use of individual swabs that might provide lower detection rates compared to pen-based oral fluids. Therefore, additional studies including pen-based oral fluids with a larger number of animals are warranted to further evaluate the efficacy and sensitivity of HM detection in oral fluids.

The PCR negative blood sample results of the 183 investigated boars from 26 different multiplier farms was somehow unexpected, as previous studies revealed a high prevalence of *M.* *suis* in sows [25–28]. However, it might be assumed that multiplier farms have a lower risk of *M. suis* introduction due the very limited purchase of animals and strict biosecurity measures.

Semen can serve as an important route for the introduction of various pathogens into a farm [29]. Up-to-date, transmission of *M. suis* via semen is thought to occur only in case of blood contamination [29, 30]. However, those studies were performed in the pre-PCR era and shedding of the pathogen in RBC-free urine, saliva and vaginal secretions has reinforced the discussion of blood-independent transmission route. Under the condition of the present study with investigating samples from one boar stud at one sampling point we were not able to detect porcine HM species in blood and semen. Nevertheless, to exclude boars and semen as potential reservoirs for HM transmission further studies including a higher number of boar studs and boars from conventional farms are certainly needed.

## Conclusion

In conclusion, our results indicate that blood independent shedding routes are unlikely to play a major epidemiological role under field conditions. In addition, the results of our experimental study did not confirm the possibility of an oral transmission for *M. suis* via infected blood. Despite several benefits over the more invasive blood sampling, individual saliva samples might not represent an appropriate sample type for the detection of *M. suis*.

## Materials and methods

### Experimental design

For experimental infection, a splenectomised pig model was used [10, 17]. The experimental protocol was approved by the Government Office of Upper Bavaria, Munich (authorization reference number 55.2-1-54-2532-87-12). A total of 14 piglets at the age of 28-days originating from the same *M.* *suis* negative farm were included in the study. The *M.* *suis* negative status was confirmed by qPCR as previously described [13, 31]. One week after placement, each piglet was splenectomised according to the protocol of Heinritzi [17]. For experimental studies with *M.* *suis* splenectomy is usually performed since the absence of the spleen reduces the incubation period, exacerbates the clinical signs of disease and enhances the replication rate of the pathogen within the host animal [10].

One week after splenectomy, piglets were randomly assigned into two groups (group A: n = 7; ID 33, 34, 37, 64, 65, 55, 69; group B: n = 7; ID 23, 31, 32, 71, 73, 74;) for experimental infection. The previously described *M.* *suis* field strain K323/13 [12] was used as inoculation strain.

Piglets of group A were inoculated orally, piglets of group B subcutaneously with *M.* *suis* containing blood (1.5 mL; 2.0 × 10^7^ *.M*. *suis*/mL). Daily clinical observation, treatment and determination of the experiment were performed as previously described [12]. Briefly, the clinical scoring system shown in Table 2 was used for daily observation of animals. Upon acute IAP attack, which is delineated by three clinical score points, animals were treated with oxytetracycline (20 mg/kg body weight/24 h, i.m.) and glucose (35 g/L, oral). Additionally, Metamizole (30 mg/kg body weight) was administered intramuscularly if the body temperature exceeded 42 °C. The termination criteria of the experiment were defined as follows: a clinical score of > 3 remaining constant over 48 h despite antibiotic treatment, sustained fever of > 40 °C and impaired general health and anorexia. On reaching these criteria, the affected animal was euthanized by intravenous pentobarbital injection (45 mg/kg body weight).

**Table 2:** Clinical scoring system used for daily animal observation during experimental *M. suis* infection (in accordance with Stadler et al. [12])

Score points	Ears	Skin	Body temperature	Behavior	Feed intake	Respiration
0	No alterations	No alterations	< 40 °C	No alterations	No alterations	No alterations
1	Mild cyanosis	Moderate pallor	40–42 °C	Reduced	Reduced	Mild dyspnoe
2	Moderate cyanosis and necrosis	Generalised petechiae	> 42 °C	Apathy	Anorexia	Severe dyspnoe
3	–	Icterus	–	–	–	–

EDTA-anticoagulated blood samples (puncture of *V. jugularis*), urine and saliva samples were collected every two days for the first 8 days post infection (DPI) and subsequently once a week until the end of the trial on 90 DPI. Furthermore, individual samples were taken on additional time points when clinical signs exacerbated in the affected animal.

Individual saliva collection was performed without restraining of the animals. Saliva samples were obtained as described elsewhere [32, 33]. Briefly, the pigs were allowed to chew on a cotton swab with the help of a metal rod, until the swab was thoroughly soaked with saliva (Salivette^®^, Sarstedt, Aktiengesellschaft and Company, Nümbrecht, Germany). After sample collection, the swab was placed in a sealed plastic vial and was centrifuged at 4000×*g* for 8 min. Urine samples were taken by spontaneous urination in sterile tubes. Saliva and urine samples were stored at − 80 °C until further processing.

Gross-necropsy and histopathological examination was performed of all animals. as previously described [12]. In brief, tissues were fixed in paraformaldehyde, embedded in plastic and were stained for Giemsa and haematoxyline-eosin-phloxin.

### Field samples

Blood, saliva and urine samples of 150 sows were available from a previous study (stored at − 80 °C) [34] (authorization reference number 55.2-154-2532.2-16-13) and collected as described above. The samples originated from 15 piglet producing farms in Southern Germany. The chosen farms were preselected as being positive for *M.* *suis* by detection of *M. suis* in blood samples of sows by qPCR. In total, 148 saliva samples (8–10 saliva samples and corresponding blood samples per farm) from 15 *M. suis* positive farms and 47 urine samples (1–8 urine samples and corresponding blood samples per farm) from 11 M*. suis* positive farms were investigated for the presence of *M. suis* by qPCR. A minimum of 10% positive results were assumed for the statistical analysis (Binomial test and Power calculation), which was calculated with BIAS for Windows 11.01 (Epsilon-Verlag, Frankfurt; Germany).

Additionally, semen and EDTA-blood samples obtained from a German boar stud during regular on-farm health monitoring were also included in the present study. In total semen and EDTA-samples of 183 boars originating from 26 different multiplier farms were collected. The boars’ age ranged from 9 to 77 months. Samples were collected within one day and stored at -80 °C until further processing. Investigations were approved by the ethical commission of the veterinary faculty of the LMU, Munich (authorization reference number: 245-17-12-2020).

### Methods

DNA was extracted from EDTA-anticoagulated blood, urine, and saliva samples as described previously [11, 13]. Urine samples were further tested with Servotest^®^ 5 + NL stripes (Servoprax, Wesel, Germany) for RBC residues. Semen samples were pooled to five and DNA was extracted by using the QIAamp^®^ DNA mini kit (Qiagen GmbH, Hilden, Germany) according to the manufacturer’s instructions. DNA samples were investigated for *M.* *suis* and ‘*Ca*. M. haemosuis’ by qPCR as previously described [6, 13, 31]. Briefly, the following primers targeting the *msg1* gene of *M. suis* (msg1-Fw 5′-ACAACTAATGCACTAGCTCCTATC-3′ and msg1-Rv 5′-GCTCCTGTAGTTGTAGGAATAATTGA) and the gap gene of ‘*Ca*. M. haemosuis’ (CMhsuisF 5′-TGCTTTGGCTCCTGTGGTTA-3′ and CMhsuisR 5′- GCAGCAGCACCTGTAG AAGTA-3′) were used. The 178 bp fragment (*M.* *suis*) and the 177 bp fragment (‘*Ca*. M. haemosuis’) were each detected and quantified using the StepOne™ System (Applied Biosystems^®^). QPCR was carried out with Fast SYBR^®^ Green PCR and the following cycling conditions: 95 °C for 10 min followed by 40 cycles of 95 °C for 15 s and 60 °C for 30 s and subsequent melting curve analysis.

## Data Availability

All datasets used in this study are available from the corresponding author on reasonable request.
